# Iso-C3D navigation assisted pedicle screw placement in deformities of the cervical and thoracic spine

**DOI:** 10.4103/0019-5413.62083

**Published:** 2010

**Authors:** Vinod V Rajan, Vijay Kamath, Ajoy Prasad Shetty, S Rajasekaran

**Affiliations:** Department of Orthopaedics and Spine Surgery, Ganga Hospital, 313, Mettupalayam Road, Coimbatore – 641 011, Tamil Nadu, India

**Keywords:** Navigation, pedicle screw fixation, thoracic and cervical deformity

## Abstract

**Background:**

Pedicle screw instrumentation of the deformed cervical and thoracic spine is challenging to even the most experienced surgeon and associated with increased incidence of screw misplacement. Iso-C3D based navigation has been reported to improve the accuracy of pedicle screw placement, however, there are very few studies assessing its efficacy in the presence of deformity. We conducted a study to evaluate the accuracy of Iso-C3D based navigation in pedicle screw fixation in the deformed cervical and thoracic spine.

**Materials and Methods:**

We inserted 98 cervical pedicle screws (18 patients) and 242 thoracic pedicle screws (17 patients) using Iso-C3D based navigation for deformities of spine due to scoliosis, ankylosing spondylitis, post traumatic and degenerative disorders. Two independent observers determined and graded the accuracy of screw placement from postoperative computed tomography (CT) scans.

**Results:**

Postoperative CT scans of the cervical spine showed 90.8% perfectly placed screws with 7 (7%) grade I pedicle breaches, 2 (2%) grade II pedicle breaches and one anterior cortex penetration (< 2mm). Five lateral pedicle breaches violated the vertebral artery foramen and three medial pedicle breaches penetrated the spinal canal; however, no patient had any neurovascular complications. In the thoracic spine there were 92.2% perfectly placed screws with only six (2%) grade II pedicle breaches, eight (3%) grade I pedicle breaches and five screws (2%) penetrating the anterior or lateral cortex. No neuro-vascular complications were encountered.

**Conclusion:**

Iso-C3D based navigation improves the accuracy of pedicle screw placement in deformities of the cervical and thoracic spine. The low incidence of pedicle breach implies increased safety for the patient.

## INTRODUCTION

Pedicle screw instrumentation is the preferred method of posterior stabilization of the spine especially in the presence of deformity.^1^–^3^ It has the advantages of three column fixation, improved coronal, sagittal, and rotational correction, lower incidence of implant failures, pseudoarthrosis and fewer requirements of postoperative bracing when compared with conventional hook and wire constructs.^4^–^6^

However, pedicle screw instrumentation of the deformed thoracic^3^,^7^ and cervical spine is associated with a high rate of pedicle breach (up to 43% for thoracic spine^7^ and 12% for cervical spine^8^) and though the ensuing complication rate is low^7^–^9^ the potential for disastrous neurovascular injury, remains. Other complications such as loss of fixation of the curve, progression of deformity and pseudoarthrosis may ensue from poor fixation.

Computer navigation has been found to improve the accuracy of pedicle screw fixation in deformed as well as non deformed spine.^7^,^10^–^16^ Most of these studies,^10^,^14^,^15^ however, have the drawbacks of including deformities of the lumbar spine or having deformities from only a small subsection of the study population. Hence these studies are of limited value in assessing the true efficacy of computer navigation. The narrow diameter of the thoracic and cervical pedicles and the proximity of the cord and vertebral artery (in the cervical spine) make pedicle screw insertion challenging. The presence of deformity adds to the difficulty and hence the true efficacy of computer navigation can be gauged by assessing the accuracy of pedicle screw placement in cervical and thoracic spine deformity correction. There are very few studies^11^–^13^,^17^ reporting on the efficacy of computer navigation in the deformity correction. Hence we conducted a prospective study to evaluate the accuracy of screw placement using Iso-C3D navigation in cervical and thoracic spine deformities.

## MATERIALS AND METHODS

Seventeen patients with thoracic spine deformity and 18 with cervical spine deformities, who were treated by pedicle screw fixation using Iso-C3D based navigation, were included in the study. Patients with scoliosis of more than 100° or kyphosis exceeding 90° as well as obese patients (weight of more than 100 kg) were excluded from the study group as in these cases it is impossible to centralize the anatomic area of interest in the arc of the movement of the Iso-C3D C-arm. The etiology of the deformities is seen in Tables 1 and 2.

**Table 1:** Etiology of thoracic spine deformities

Etiology	No. of patients (n=17)
Adolescent idiopathic scoliosis	10
Post tubercular kyphosis	3
Kyphoscoliosis due to neurofibromatosis	2
Scheurmanns disease	1
Paralytic scoliosis	1

**Table 2:** Etiology of cervical spine deformities

Etiology	No. of patients (n=18)
C1-2 instability	8
Congenital deformity	4
Sub axial instability (post traumatic, degenerative)	3
Ankylosing spondylitis with deformity	2
Ankylosing spondylitis with pseudoarthrosis	_1_

All the patients were investigated with radiographs; CT and MRI scan pre-operatively. In cervical spine, MR angiography was done to assess the course of the vertebral artery and rule out any anomalies. In thoracic spine group the mean age of the patient was 19.6±9.3 years (range 10-52 years).The mean Cobb's angle was 62.4°±6° (range 52°-86°) and mean Kyphotic angle was 36.4°±6° (range 30°-74°). In the cervical spine group, the mean age of the patient was 24.2± 6.2 years (range 10-52 years).

### Procedure

**Figure 1:**
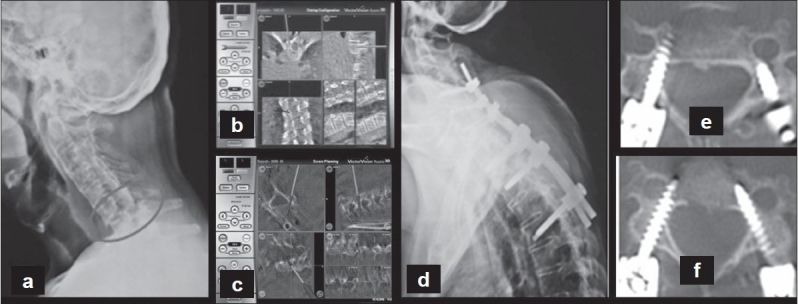
(a) Preoperative lateral radiograph of cervical spine showing a C6-7 chance fracture in a patient with ankylosing spondylitis (b, c) Intra operative navigation images of thoracic and cervical spine- pedicle entry point and trajectory and planning of screws (d, e, f) Postoperative radiograph and CT scan showing the placement of the screws

All cases were operated in the prone position on a carbon top radiolucent table (Fahmed, Germany). After the spine was exposed posteriorly a minimally invasive reference array (MIRA) was attached to the spinous process of a vertebra caudal to the region of interest. The Siremobil Iso-C3D (Seimens, Germany) image intensifier which rotates through an arc of 190° obtained fluoroscopic images of the relevant segments of the spine. It was usually possible to include five to six levels in children and four to five levels in adults in a single fluoroscopic field. The acquired data was transferred to the computer navigation platform-Vector Vision (Spine version 2.0 Brain Lab, Germany) which reconstructed the data to provide real time intra-operative multi-planar images of the vertebra. Accuracy of the images was verified using a pointed tool navigator. The tool navigator was used along with the 3D real-time multi-planar images to determine the entry point, trajectory, length and width of the pedicle [Figure 1]. The dorsal cortex at the identified entry site was removed with a 2.5 mm burr. The pedicle was gently negotiated using a sharp pedicle finder.

The direction and depth of the pedicle finder was guided and confirmed frequently using the tool navigator. The length and size of the screws was planned using the tool and “planning a screw” option of the software. Each screw was calibrated and threaded along the pre-tapped pilot hole guided by “autopilot” images. In thinned, and deformed, and sclerosed thoracic pedicles, where transpedicular screw fixation was impossible, an in-out-in technique was used under navigation guidance. Time required for data acquisition was divided by the total number of screws inserted with the same data. This average setup time per screw added to the time taken for actual screw insertion gave the insertion time for that particular screw.

**Figure 2:**
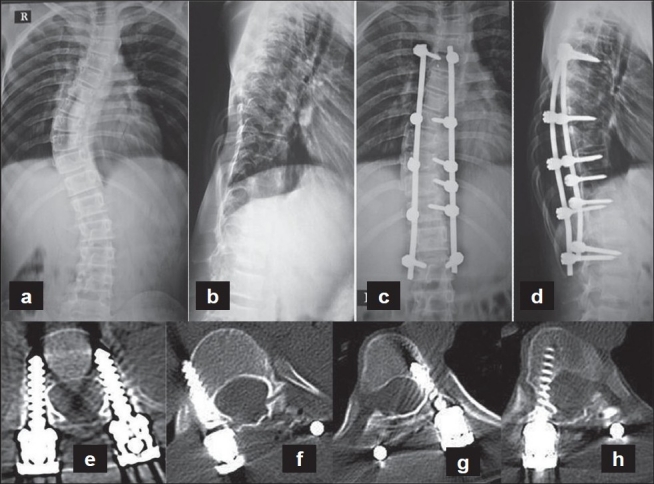
(a-d) Preoperative and post operative radiographs of an adolescent idiopathic right thoracic scoliosis. Computer assisted pedicle screw insertion was performed (e-h). Postoperative axial CT scan sections of the patient showing the placement of the screws

**Figure 3:**
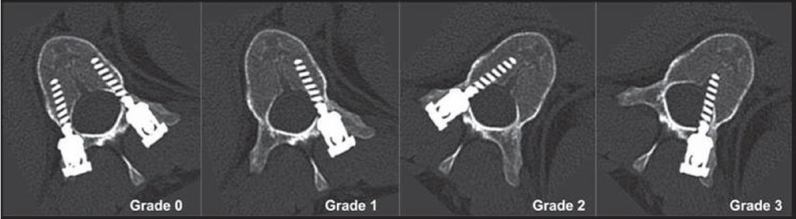
Grading used for pedicle breaches on postoperative CT scan images

Postoperative CT scans were performed using 2-mm cuts with 1-mm overlap to assess the accuracy of screw placement in all patients. The radiographs and CT scans were analyzed with respect to the breach of the pedicle wall by the screw either medially, laterally, inferiorly, or superiorly [Figure 2]. The distance of the tip of the screw from the anterior or lateral cortex of the vertebra was also measured in axial CT sections. Screw placement was graded on CT as follows [Figure 3]: grade 0, no pedicle perforation; grade 1, only the threads outside the pedicle (less than 2 mm); grade 2, core screw diameter outside the pedicle (2-4 mm); and grade 3, screw entirely outside the pedicle.

## RESULTS

A total of 242 thoracic pedicle screws and 98 cervical screws were inserted under navigation guidance. The incidence of pedicle screw breach in thoracic spine was 19/242 (7.8%). Eight patients (3%) had grade 1 pedicle breaches, six patients (2.5%) had grade 2 pedicle breaches and five (2%) had anterolateral vertebral body penetration. Of the 15 screws breaching the lateral pedicle cortex, seven with more than a 4-mm pedicle breach belonged to the planned in-out-in screws, hence these screws were not considered in the analysis of pedicle breaches. The average screw insertion time (includes average data acquisition time per screw) was 2.37±0.72 minutes (range 1.16-4.5) per screw. The mean data acquisition time was 24.6±6.3 minutes (range 16-33). Out of twenty four screws in patients with kyphosis four had pedicle breaches and 10 out of 208 screws in scoliotic deformity had pedicle breaches.

In patients with cervical pedicle screws there were nine pedicle breaches in ninety eight screws (9.2%). The main pedicle breach was on the lateral wall (5/98) followed by medial wall (3/98), superior pedicle (1/98) and anterolateral body breach (1/98). The grade of pedicle breach is in Table 3. Average pedicle screw insertion time in cervical spine was 2.5±0.6 min. In our series there were no incidences of grade 3 pedicle breaches, spinal cord injury or vertebral artery injury due to pedicle breach.

**Table 3:** Incidence and grade of pedicle breach

Pedicle breach		Thoracic (n=242)	Cervical (n=98)
Superior pedicle wall	Grade-1	1	1
	Grade-2	0	0
	Grade-3	0	0
Inferior pedicle wall	Grade-1	2	0
	Grade-2	1	0
	Grade-3	0	0
Medial pedicle wall	Grade-1	1	2
	Grade-2	1	1
	Grade-3	0	0
Lateral pedicle wall	Grade-1	4	4
	Grade-2	4	1
	Grade-3	7 (planned-in out in technique)	0
Anterolateral vertebral body wall	Grade-1	2	1
	Grade-2	2	0
	Grade-3	1	0

## DISCUSSION

Pedicle screw stabilization is bio-mechanically superior to other methods of spinal fixation but screw misplacement has the potential for serious complications such as spinal cord, nerve root, visceral or vessel injury.^2^,^18^–^21^ Though commonly used in the lumbar and non deformed thoracic spine, its use in the deformed thoracic and cervical spine is limited for fear of inadvertent neuro-vascular injury.^8^ The complex anatomy of the normal cervical spine including the narrow diameter of the pedicles, the variations in the saggital and axial angulations of the pedicles^22^–^25^ as well as the anatomical variations in the course^24^ and size of the vertebral artery make pedicle screw instrumentation a challenging procedure. The presence of deformity and altered anatomy (due to congenital anomalies) further adds to the difficulty.

In the thoracic spine the pedicles are narrow and the canal cord ratio of thoracic spine is low, hence the chance of a pedicle violation causing cord injury is common even in normal thoracic spine.^26^–^30^ In the presence of deformity the size and orientation of the pedicles vary considerably between the different vertebrae within the curve, and also between the concave and convex sides of the same vertebrae. The pedicles are frequently thinner and sclerosed, making canal perforation easily possible. The dura is often stretched over the pedicles on the concave side of the curve, and even minor medial violations can damage the cord.^6^–^7^,^31^–^32^ Hence pedicle screw instrumentation is challenging as there are potential risks of iatrogenic damage to neural or vascular structures.

Pedicle screw insertion is performed either by the conventional technique (using anatomical landmarks with or without fluoroscopy), foramino-laminotomy technique or computer navigation assisted. Clinical and cadaveric studies assessing the conventional technique of pedicle screw placement have reported pedicle violation rates ranging from 5.5 to 54.7% for lumbar and thoracic region with neurologic sequelae ranging from 0 to 7%.^7^,^10^,^21^,^33^ In the cervical spine it ranges from 6.7 to 12% with neurological sequelae ranging from 0 to 1.7%.^8^,^34^–^36^ The foramino- laminotomy technique has been be used to increase the accuracy of screw placement, however, pedicle violation rates up to 15.9% in the thoracic spine and 55% in the cervical spine have been reported.^36^–^37^ The open-lamina technique requires increases surgical exposure of the epidural space, which can potentially result in increased operative time, risk of dural tear, and blood loss.^37^

Computer navigation is commonly used to guide pedicle screw insertion and studies comparing pedicle screw placement using conventional and computer navigation have demonstrated the superior accuracy of this technology.^8^,^10^,^12^,^13^,^17^,^36^ However, there are very few studies^11^–^13^,^17^ documenting the efficacy of computer navigation in placement of pedicle screws in the deformed cervical and thoracic spine.

### Accuracy of screw placement in thoracic spine deformities

Belmont *et al*.^31^ reported the clinical accuracy of thoracic pedicle screws in 40 patients of scoliosis and kyphosis, demonstrating a 43% of screw perforation rate. Liljenqvist *et al*.^3^ reported on 33 patients of thoracic idiopathic scoliosis and demonstrated a 25% of perforation rate. In both the studies,^3^,^31^ despite the high screw perforation rate, there were no neurovascular or pulmonary complications. A study by Laine *et al*.^14^ on 100 patients, including small numbers of spinal deformity demonstrated that the screw perforation rate signifcantly decreased from 13.4 to 4.6% with use of computer navigation in thoracic and lumbar spine. In a comparative study on the accuracy of pedicle screw placement in scoliosis between conventional fuoroscopic and computer-assisted surgical techniques, Kotani *et al*.^12^ observed a perforation rate of 11% in the conventional group and 1.8% in the navigated group. The results of the present study (7.8% breaches) are lower than those reported using the conventional technique and demonstrate the improved accuracy of computer navigation in the insertion of pedicle screws. The in-out-in technique was used to insert seven screws as a planned procedure. This was done as the pedicles were sclerosed and too thin to accept a screw without a pedicle breech. Here the screw trajectory was not an accidental breach but a well planned trajectory aimed at a secure position of the screw in the body, hence these screws were not included in the accidental breach category.

### Accuracy of screw placement in cervical spine deformities

In the present study of the cervical spine region there were nine cases of pedicle breaches (9.2%), but none of the cases had vertebral artery, spinal cord or nerve root injuries. Even when lateral wall perforation occurred, no complications involving the vertebral artery were clinically apparent. It is theorized that the vertebral artery does not occupy the whole of the foramen transversarium, hence minimal violations of the foramen transversarium may not be as risky as was initially thought.^35^–^36^ Richter *et al*.^38^ reported 8.6% of pedicle screw perforation with conventional technique and 3% with CT based navigation assisted pedicle screw fixation. Though Abumi *et al*.^34^ reported only a 6.7% misplacement rate (out of 669 cervical pedicle screws) when using a conventional screw insertion technique this data may not be applicable to other spine surgeons with less experience in cervical pedicle screws. In a comparative study between the conventional and computer navigated technique for cervical pedicle screw insertion by Kotani and Abumi *et al*.,^12^ the rate of pedicle wall perforation was significantly lower in the computer-assisted group than that in the conventional group (1.2 and 6.7%, respectively; *P*<0.05). The screw trajectory in the horizontal plane was significantly closer to the anatomical pedicle axis in the computer-assisted group compared with the manual insertion group (*P*<0.05). Complications such as neural damage or vascular injury were not demonstrated in the computer-assisted group (compared with 2% in the manual insertion treatment group). This further underlines the fact that computer navigation improves the accuracy of screw placement. The high rate of pedicle breaches seen in the present series may be due to the presence of congenital anomalies.

Even though there are many advantages of computer assisted surgery, the surgeon must be prepared to proceed, in rare instances, without image guidance. In case of severe deformity it is difficult to centralize the patient in both antero-posterior and lateral view in C arm. In gross obesity the arc of C arm cannot move freely around the patient. In case of osteoporosis the image quality will be poor. Moreover, navigation is a computer and software program and the system can crash at any time. Like all image-guidance methods, the Iso-C3D is not a substitute for anatomic knowledge of the spine. The results of this analysis indicate that image guided surgery systems should not be used as a substitute for surgical judgment and experience, but rather as a tool to complement the surgeon.

## CONCLUSION

Intra operative Iso-C3D fluoroscopy based navigation improves the accuracy of pedicle screw insertion and reduces the pedicle screw perforations in deformities of cervical and thoracic spine. Its use in very severe deformities (scoliosis >90° and kyphosis >90°) is limited.
